# Deciphering Transcriptional Dynamics In Vivo by Counting Nascent RNA Molecules

**DOI:** 10.1371/journal.pcbi.1004345

**Published:** 2015-11-06

**Authors:** Sandeep Choubey, Jane Kondev, Alvaro Sanchez

**Affiliations:** 1 Department of Physics, Brandeis University, Waltham, Massachusetts, United States of America; 2 Rowland Institute at Harvard, Harvard University, Cambridge, Massachusetts, United States of America; ETH Zurich, SWITZERLAND

## Abstract

Deciphering how the regulatory DNA sequence of a gene dictates its expression in response to intra and extracellular cues is one of the leading challenges in modern genomics. The development of novel single-cell sequencing and imaging techniques, as well as a better exploitation of currently available single-molecule imaging techniques, provides an avenue to interrogate the process of transcription and its dynamics in cells by quantifying the number of RNA polymerases engaged in the transcription of a gene (or equivalently the number of nascent RNAs) at a given moment in time. In this paper, we propose that measurements of the cell-to-cell variability in the number of nascent RNAs provide a mostly unexplored method for deciphering mechanisms of transcription initiation in cells. We propose a simple kinetic model of transcription initiation and elongation from which we calculate nascent RNA copy-number fluctuations. To demonstrate the usefulness of this approach, we test our theory against published nascent RNA data for twelve constitutively expressed yeast genes. Rather than transcription being initiated through a single rate limiting step, as it had been previously proposed, our single-cell analysis reveals the presence of at least two rate limiting steps. Surprisingly, half of the genes analyzed have nearly identical rates of transcription initiation, suggesting a common mechanism. Our analytical framework can be used to extract quantitative information about dynamics of transcription from single-cell sequencing data, as well as from single-molecule imaging and electron micrographs of fixed cells, and provides the mathematical means to exploit the quantitative power of these technologies.

## Author Summary

Gene expression starts with transcription, a multi-step process that produces an RNA molecule that is complementary to the gene. Cells often control the amount of gene expression by controlling the amount of RNA produced through interactions between regulatory DNA and proteins involved in transcription. While the identity of the molecules that take part in this regulatory process is known for a number of different genes, their dynamics in cells is still poorly understood. We show theoretically that the cell-to-cell variability in the number of nascent RNA molecules, those still in the process of being synthesized by the RNA polymerase, carries the signature of transcriptional dynamics in cells. We analyze published nascent RNA distributions for a set of yeast genes and show that the data is inconsistent with a single-step model of transcription initiation. Instead we propose a coarse-grained model where initiation happens not in one but in two sequential steps. Our analytical framework can be used to extract quantitative information about the dynamics of transcription from single-cell sequencing data, as well as from single-molecule imaging and electron micrographs of fixed cells.

## Introduction

Transcription is a multi-step process that leads to the production of messenger RNA (mRNA) molecules from its DNA template. Genetic experiments on cells have identified the key molecular components of transcription, while biochemical studies with purified components have uncovered the basic mechanisms governing their dynamics and interactions in vitro. Still an important question that remains is whether the same mechanisms are also operational in cells. One approach to unraveling the mechanisms of transcription in cells is to measure the outputs of this process, either the proteins that correspond to the genes being transcribed, or the actual mRNA molecules. This idea has motivated numerous experiments that count protein [1–3], and mRNA [4–6] molecules in single cells. The measured steady state distribution of these molecules in a clonal cell population can then be used to infer the dynamics of transcription [4,5]. For instance, analysis of the steady state distributions of cytoplasmic mRNA in yeast for a number of different genes, have suggested that yeast genes may fall into two different classes: those that are transcribed in random uncorrelated events clearly separated in time and without any transcriptional memory [4] (this is often referred to as Poissonian transcription), and those that are transcribed in bursts caused by the promoter switching slowly between an active state and an inactive state (this is often referred to as Bursty-transcription [7,8]).

While this approach to deciphering transcriptional dynamics *in vivo* by counting cytoplasmic RNA in single cells has led to important insights, a key limitation is that processes that are downstream from transcription initiation can mask the signature of transcriptional dynamics in measurements of the cell-to-cell variability of mRNA and protein abundances. A striking example of this is the recent finding that spatial and temporal averaging, i.e., the process of accumulation and diffusion of mRNA transcripts during nuclear cycles, significantly reduces the variability in mRNA copy number expected from stochastic transcription initiation [9]. In addition, effects such as mRNA transport out of the nucleus, mRNA processing, and nonlinear mRNA degradation [10–14] can also in principle affect the level of variability of cytoplasmic mRNA. All of these non-transcriptional sources of variability may propagate to the protein level as well, affecting the cell-to-cell fluctuations in protein copy number, which is also affected by the stochastic nature of translation. Finally, it has been recently shown that partitioning of both mRNA and protein molecules during cell division [15–17] can generate distributions in their abundances similar to those that would be generated by stochastic transcription and translation. Therefore, the cell-to-cell variability of both protein and cytoplasmic mRNA copy number do not necessarily reflect transcriptional dynamics alone but are determined by a combination of stochastic processes of which transcriptional dynamics is just one component [18].

One alternative to analyzing steady state mRNA and protein distributions, has been to directly image transcription in real time using fluorescently labeled RNA-binding proteins that associate with nascent RNA, which is still in the process of being assembled at the gene by the RNA polymerase [19–23]. When applied to *E*. *coli*, *Dyctostelium* or animal cells this technique revealed widespread transcriptional bursting consistent with the mechanism of transcription initiation where the promoter switches between an active state and an inactive state [7]. In contrast, in experiments on two constitutive and cell-cycle activated genes in *S*.*cerevisiae*, Larson et al. [23] found that the transcription initiation process is dominated by one rate limiting step. In spite of the great promises of this approach, it is technically challenging and still remains in its infancy.

**Fig 1:**
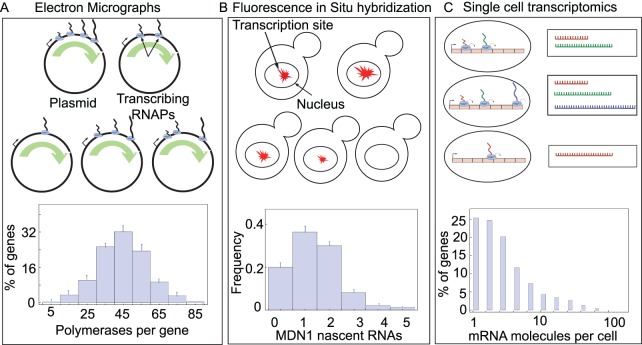
Experimental methods to count nascent RNA (transcribing RNA polymerase molecules). (A) Electron micrographs of intact chromosomes extracted from cells provide images of transcribing polymerases along a gene (also referred to as Miller spreads). This method allows one to count the number of RNAP molecules that are actively transcribing a gene of interest across a population of genetically identical cells. Histograms for the distribution of number of Pol I molecules is shown along rDNA, for a wild type yeast cell (adapted from [67]). (B) Fluorescence in Situ Hybridization (FISH) in single cells provide the intensity of the transcription site, which can then be used to count the number of nascent RNAs for a particular gene. Histogram for the nascent RNA distribution is shown for MDN1 gene in yeast (adapted from [25]) (C) Such measurements can be used to count the number of nascent RNA transcripts using the fact that the length of nascent RNA transcripts are shorter than the mRNA transcripts. Histograms for mRNA distribution [68] in ES cells is shown.

Lately, a score of experimental papers have reported measurements of distributions across a clonal cell population of nascent RNA transcripts at a single gene, using single-molecule fluorescence in-situ hybridization (FISH) [4,24–26] (Fig 1B). These experiments reveal the number of RNA polymerases engaged in transcribing a single gene in a single cell at a specific instant in time. This information can also be obtained from so-called Miller spreads (electron micrographs of intact chromosomes extracted from cells) which provide images of transcribing polymerases along a gene [27–31] (Fig 1A). Perhaps more importantly, single-cell whole genome RNA sequencing is slowly but steadily being developed and turned into a quantitative technique, one which will be able to provide a snapshot of the number of RNA polymerase molecules engaged in the transcription of every gene in the cell at a given instant in time (Fig 1C) [32–36]. Counting nascent RNAs (or the number of transcribing polymerases) provides a more direct readout for the transcriptional dynamics at the promoter within the short window of time required for an RNA polymerase molecule to complete elongation (for a typical gene in yeast the elongation time is of the order of few minutes [4]). As such, this experimental approach is not affected by the aforementioned stochastic processes that contribute to cytoplasmic mRNA and protein fluctuations. Indeed, as mentioned above, strong discrepancies between cytoplasmic and nascent mRNA distributions have been recently found in Drosophila embryos [9]. Below we also demonstrate similar discrepancies in yeast by analyzing published data obtained from counting nascent and cytoplasmic mRNA in single cells.

It is thus starting to become possible to obtain quantitative measurements of the distribution of nascent RNA or, what is the same, of the number of transcribing polymerases per gene. In spite of its many advantages, the potential of nascent RNA distributions has not been fully exploited (for a notable exception see [37]) due to the lack of mathematical formalisms that allows one to connect molecular mechanisms of transcription initiation and elongation with measured nascent RNA distributions. One of the key results that we report here is the development of such formalism. In particular, we show how to compute the mean and variance of the distribution of nascent RNAs for an arbitrary mechanism of transcription initiation and stochastic elongation. The results of these calculations provide the tools to extract information about transcriptional dynamics from experimentally determined nascent RNA distributions. We demonstrate the usefulness of our method by analyzing published nascent RNA distributions for a set of constitutively expressed yeast genes [25]. We find that all of these yeast genes have similar average initiation rates. We also find that initiation of transcription of these yeast genes is a two-step process, where the average durations of the two steps are equal. This is in sharp contrast to the conclusion that was reached for some of these genes by counting cytoplasmic mRNAs, namely that transcription initiation is dominated by one rate limiting step [25]. By analyzing the nascent RNA distribution, we are able to reach a level of kinetic detail, particularly fast processes, which are obscured at the level of cytoplasmic RNA. While the molecular identities of the two steps leading to transcription initiation remain unknown, our results point to the existence of multiple transcription initiation steps in vivo. It is worth emphasizing that multiple initiation steps of similar duration lead to a reduction of fluctuations in the number of nascent RNAs in a cell, when compared to those produced by single-step initiation.

## Results

### Stochastic model of transcription initiation and elongation

In order to connect mechanisms of transcription initiation with nascent RNA distributions, we consider a model of transcriptional dynamics with an arbitrarily complex initiation mechanism followed by an elongation process. We describe both processes using chemical master equations. This approach is inspired by the work of Kepler et al. [38] who computed the moments of the mRNA distribution for a promoter, where it switches between an active and an inactive state. We have previously developed this method further to compute the moments of mRNA and protein distributions for arbitrarily complex promoters that can switch between multiple states, each state leading to transcript production at a particular rate [8,39–42]. Here we implement the same master equation approach to compute the first and second moments of the nascent RNA distribution. A new element in our analysis is the explicit inclusion of the stochastic elongation process, which predicts that the nascent RNA distributions depend on the length of the gene being transcribed, for which we find confirmation in published data. This dependence of the distribution of nascent RNAs on gene length has also been described recently in [37]. Our theory also suggests new experimental approaches to deciphering the dynamics of transcription initiation in vivo, in which the length of the transcribed gene is varied and the effect on the number of nascent RNAs is measured.

**Fig 2:**
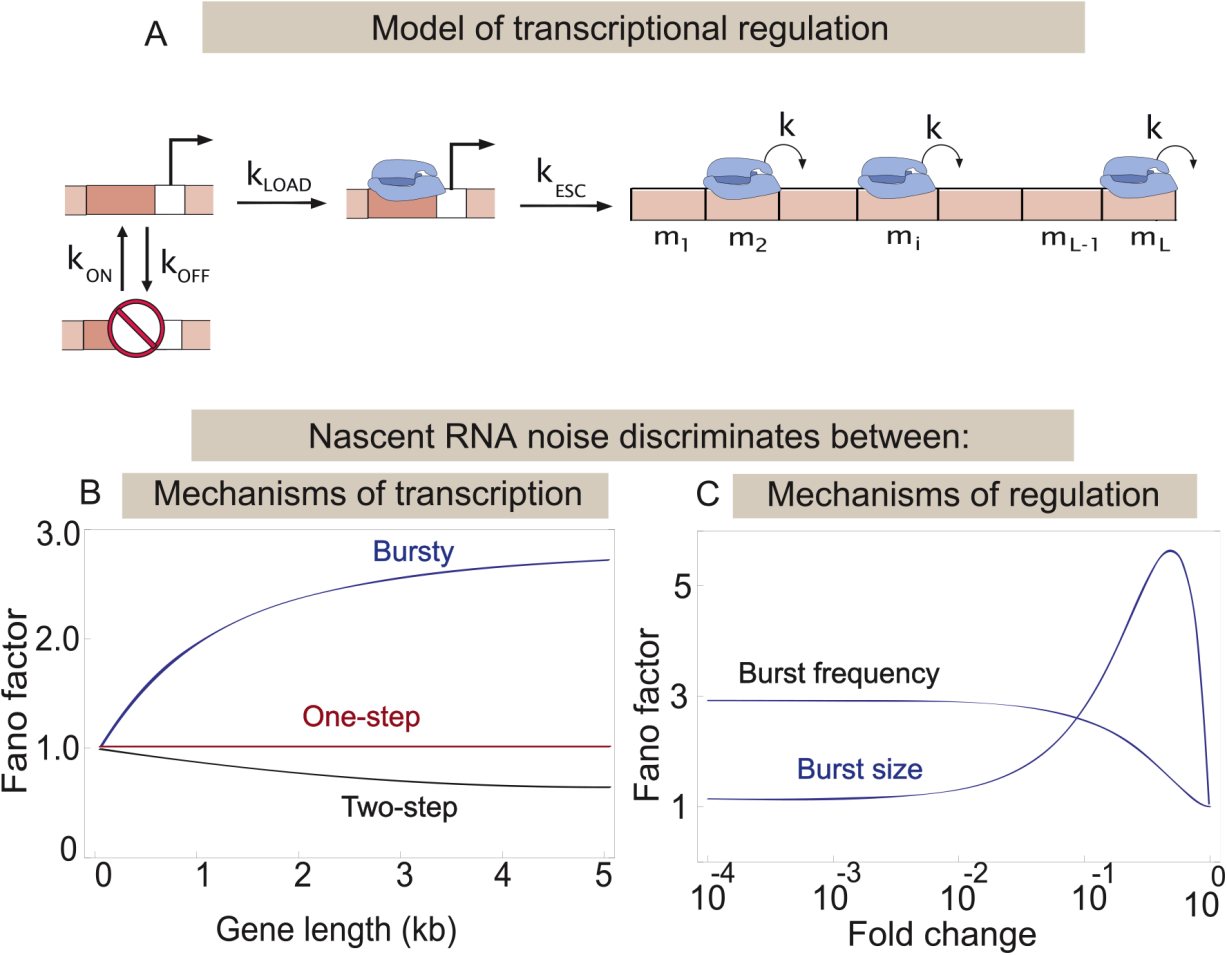
(A) Model of transcriptional regulation. The promoter switches between two states: an active and an inactive one. The probability per unit time of switching from the active state to the inactive state is *k*_*OFF*_, and from the inactive to the active state is *k*_*ON*_. From the active state transcription initiation occurs in two sequential steps: the formation of the pre-initiation complex at the promoter proceeds with rate *k*_*LOAD*_ after which the RNA polymerase escapes the promoter at a constant probability per unit time *k*_*ESC*_. Once on the gene the polymerases move from one base pair to the next with a rate *k*, until they reach the end of the gene and they fall off with the same rate. From this model we compute the mean and the variance of the number of RNA polymerases, present on the gene in steady state, as a function of all the rates and the length of the gene *L*. This calculation is aided by introducing the *m*_*i*_ variables for every base, which keep track of the number of polymerases at that base. **(B) Noise profile for different models of transcription initiation.** From the master equation of the model described in (A) we computed the Fano factor of the nascent RNA distribution as a function of the length of the gene being transcribed, for the three different models of transcription initiation: one-step (red), "bursty"(blue), and two-step initiation (black). The three different models give qualitatively distinct predictions. To illustrate this point for the "bursting" model we use the following parameters: *k*_*OFF*_ = 5/min, *k*_*ON*_ = 0.435/min, *k* = 0.8kb/min, *k*_*LOAD*_ *=* 5/min and *k*_*ESC*_ *=* 0/min, which are characteristic of the PDR5 promoter in yeast, as reported in [4]. For the two-step model we use *k*_*LOAD*_ *=* 0.14/min, *k*_*ESC*_ *=* 0.14/min, *k*_*OFF*_ = 0/min, *k*_*ON*_ = 0/min, *k* = 0.8kb/min, characteristic of MDN1 promoter, which we find by analyzing the data reported in [25]. For the one-step model, we use *k*_*LOAD*_ *=* 0.09/min, *k*_*ESC*_ *=* 0/min, *k*_*OFF*_ = 0/min, *k*_*ON*_ = 0/min, *k* = 0.8kb/min, which are characteristics of the yeast gene RPB1, obtained by analyzing the data published in [25]. **(C) Noise profiles for different regulatory mechanisms.** In the "bursting" model of transcription, the transcriptional output can be modulated either by changing the burst size or the burst frequency, which in the model can be achieved by tuning *k*_*OFF*_ or *k*_*ON*_. The Fano factor for the nascent RNA distribution obtained from burst size and burst frequency mechanisms of regulation are plotted as a function of the fold change in mean. (i.e., the mean of the distribution normalized by the maximum mean number of nascent RNAs in the cell, which is obtained when there is no transcriptional regulation and the promoter is always active). Clearly the two modes of regulation give qualitatively distinct predictions for the noise profile. (To illustrate this point we use the following parameters: *k*_*OFF*_ = 5/min, *k*_*ON*_ = 0.435/min, *k* = 0.8kb/min, *L* = 4436 bps, *k*_*INI*_ *=* 5/min, which were reported for the PDR5 promoter in yeast [4].)

To describe the transcription initiation process we focus on promoter dynamics. (Here we use the term promoter to denote the stretch of regulatory DNA that controls the initiation of transcription of a specific gene.) The promoter switches between different states as different transcription factors bind and fall off their respective binding sites, causing the effective initiation rate to fluctuate. We assume that after initiation, each RNA polymerase (RNAP) moves along the gene by stochastically hopping from one to the next base at a constant probability per unit time (Fig 2A). Our model assumes that transcription initiation timescales are much slower than the elongation timescale and hence RNAPs do not interfere with each other while moving along the gene. This approximation is reasonable for all but the strongest promoters characterized by very fast initiation [43,44]. We demonstrate this explicitly using numerical simulations [45,46] which include a detailed model of transcription elongation that takes into account excluded-volume interaction between adjacent polymerases (i.e. “traffic” as defined in previous work [43]), as well as ubiquitous RNAP pausing [43,47] (please see S1A Fig). The agreement between analytical results based on our simple model and the stochastic simulations of the more realistic model that incorporates traffic jams and pausing of RNAPs only starts to break down when the initiation time scales become comparable to the elongation time scales (please see S1C and S1D Fig). We conclude that for typical rates reported for RNAP elongation and pausing the simple model of transcription adopted here reproduces the first two moments of the nascent RNA distribution with deviations from those obtained from the more realistic model that are less than 10% as long as initiation of transcription is slower than 30 initiations/min. All the initiation rates that have been reported so far from in vivo measurements are slower [4,19,23], with important exceptions such as the ribosomal promoters [43,44].

Our model does not explicitly include the rate of termination at which the RNAP departs the last base of the gene. The genes [25] that we analyze have an average initiation rate of the order of *k*_*INI*_ = 0.145±0.025/min. Hence even for a termination rate of the order of 1/min [23], the variance and mean of the nascent distribution won’t be affected for these genes. Another simplifying assumption that we make is that we place no restriction on the number of transcribing RNAPs that can occupy a given base (in reality at any given instant the number is zero or one). This is equivalent to assuming that the occupancy of any given base of the gene by a transcribing polymerase is much less than one, which holds when the initiation time scale is much slower than the elongation time scale. Hence, despite its simplicity, the model of transcription initiation and elongation we adopt here should apply to most genes.

In order to compute the first two moments of the nascent RNA distribution for an arbitrary transcription initiation mechanism, we consider a promoter that can exist in *N* possible states. The rate of transition from the *s*-th to the *q*-th state is *k*_*s*,*q*_, and the rate at which RNAP initiates transcription from the *s-th* promoter state is *k*_*s*,*ini*_. Following the initiation process, every RNAP moves along the gene (elongates) by hopping from one base to the next with a probability per unit time *k*, which is equal to the average rate of elongation. The number of RNAP molecules, which is the same as the number of nascent RNAs, at the *i-*th base pair is denoted by *m*_*i*_. Hence the number of nascent RNAs (*M*) along a gene whose length is *L* bases, is given by, $M=\sum_{i=1}^{L}m_{i}.$ As remarked earlier we do not consider the processes of transcription termination and mRNA release, as they tend to be fast on the time scales set by initiation and elongation. However these can be easily incorporated into the model. (For the mathematical details please see the S1 Text.) The state of the combined promoter+RNA system is described by (*L*+1) stochastic variables: the number of nascent RNAs (*m*_*1*,…,_ *m*_*L*_) at every base along the gene, and the label *s*, characterizing the state of the promoter. Hence, the probability distribution function that characterizes the promoter+RNA system is given by *P*(*s*,*m*_1_,…,*m*_*L*_). To stream-line the mathematics we define the following probability vector:

$$\vec{P}(m_{1},\ldots ,m_{L})=(P(1,m_{1},\ldots ,m_{L}),P(2,m_{1},\ldots ,m_{L}),\ldots ,P(s,m_{1},\ldots ,m_{L})).$$ (1)

The time evolution for this probability vector can be described by a set of chemical master equations, which can be written in compact, matrix form as

$$\begin{array}{l} \frac{d\vec{P}(m_{1},\ldots ,m_{L})}{dt}=(\hat{K}-\hat{R}-\hat{\Gamma }\sum_{i=1}^{L}m_{i})\vec{P}(m_{1},.,m_{i},.,m_{L})+\hat{R}\vec{P}(m_{1}-1,\ldots ,m_{L})\\ \;+\sum_{i=1}^{L-1}k(m_{i}+1)\hat{\Gamma }\vec{P}(m_{1},.,m_{i}+1,m_{i+1}-1,.,m_{L})+k(m_{L}+1)\;\hat{\Gamma }\vec{P}(m_{1},\ldots ,m_{L}+1). \end{array}$$ (2)

In Eq (2), we define the following matrices: $\hat{K}$, which describes the transition between different promoter states, and whose elements are *K*_*qs*_ = *k*_*q*,*s*_ if *q≠s* and $K_{ss}=-\sum_{q}k_{q,s}$. $\hat{R}$ is a matrix that contains the rates of initiation from different promoter states. In the case of one-step initiation it is diagonal with the diagonal elements equal to the rates of initiation from different promoter states. In the case of two-step initiation this matrix is off-diagonal owing to the fact that the promoter state changes after initiation (for details please see the S1 Text). $\hat{\Gamma }$ is also diagonal and its elements represent the hopping rate for the polymerase from one base pair to the next, i.e., Γ_*sq*_ = *k δ*_*s*,*q*_.

We limit our calculation to the steady state nascent RNA distribution for which the left hand side of Eq (2) is set to zero. To obtain the first and second moments of the number of nascent RNAs, $M=\sum_{i=1}^{L}m_{i}.$ in steady state we use Eq (2) to compute the quantities 〈*m*_*i*_〉 and 〈*m*_*i*_ *m*_*j*_〉 for all *i*,*j* ≤ *L*. Even though the random variables *m*_*i*_ for different bases *i* on the gene are mutually dependent, we end up deriving a set of linear equations for 〈*m*_*i*_〉 and 〈*m*_*i*_ *m*_*j*_〉 (Please see the S1 Text.) We find that these equations for the moments close, in other words they do not depend on any further, higher moments of the *m*_*i*_’s. These linear equations can then be solved to obtain exact expressions for the first two moments of *M* as a function of all the rates that define the molecular mechanism of initiation under investigation. (For the mathematical details please see the S1 Text.)

### Different mechanisms of transcription initiation can be discriminated by the nascent RNA distributions they produce

In order to demonstrate how the distribution of nascent RNAs at the transcription site can be used to extract dynamical information about the process of transcription initiation in vivo, we consider the canonical model of transcription shown in Fig 2A [38]. The gene can switch between two states: an active state, from which transcription initiation can occur, and an inactive state from which initiation does not occur. The two states might correspond to a free promoter and one bound by a repressor protein, or a promoter occluded by nucleosomes. In most theoretical studies to date transcription initiation from the active state was assumed to be characterized by a single rate-limiting step. Instead of initiation being a one-step process we consider the possibility that there are two rate-limiting steps involved in transcription initiation from the active state. These could represent the loading of the transcriptional machinery at the promoter [48,49] (in prokaryotes, this would correspond to the formation of open complex by RNAP [50–52]), which occurs with a rate *k*_*LOAD*_ followed by the RNA polymerase escaping the promoter into an elongation state (with rate *k*_*ESC*_).

Three different limits of our model correspond to the various scenarios that have been previously explored in the literature [4,19,53–55]. First we consider the limit when the promoter is always active (*k*_*OFF*_ → 0 in Fig 2A) and initiation is governed by a single rate-limiting step. This is a situation when one of the two kinetic steps leading up to initiation (either the assembly of the transcriptional machinery or the escape of RNA polymerase from the promoter) is much slower than the other. In this case we find that the nascent RNA distribution is characterized by a variance that is equal to the mean. In other words the Fano factor, defined as the variance divided by the mean, to characterize cell-to-cell variability is 1.The second limit of interest is when the rates of assembly of the transcriptional machinery (*k*_*LOAD*_) and promoter escape (*k*_*ESC*_) have comparable magnitudes, i.e., transcription initiation is a two-step process. In this limit, transcription initiation events are anti-correlated due to the presence of a “dead-time” or refractory period in between subsequent initiation events. The third limit of interest is the “transcriptional bursting”, when the promoter is not always active, but is slowly switching between the active and inactive states [7]

A key prediction of our model of stochastic transcription initiation and elongation, which is described in Fig 2A, is how the cell-to-cell fluctuations of the nascent RNA number depend on the length of the gene being transcribed. This dependence was also explored in [37] where the importance of the elongation rate and gene length in determining nascent RNA distributions was described, and nascent RNA distributions were used to infer the kinetic rates. Gene length is an interesting quantity to consider from the point of view of experiments, both due to the natural variation in gene length, and the ability to synthetically alter the length of the gene being expressed from a promoter of interest by genetic manipulation. Calculations of the Fano factor as a function of gene length (Fig 2B) reveal that this quantity easily discriminates between the three models of transcription initiation described above. When the gene length is small the Fano factor is close to one for all three models of initiation. As the gene length increases, the Fano factor increases above one for the “bursting” scenario, due to slow switching of the promoter between an active and an inactive state, but it decreases below one when the promoter is always active and there are two rate limiting steps leading up to elongation. Finally, in the case when initiation is dominated by one rate-limiting step and the promoter is always active, the Fano factor is equal to one, independent of gene length.

Qualitatively these results can be understood by recalling that with a single initiation step, the waiting time between initiation events is exponentially distributed. In this case the number of initiation events to occur in a time interval set by the elongation time (which is roughly equal to the number of nascent transcripts) is given by a Poisson distribution [56], for which the Fano factor is one. For two or more rate limiting steps leading to initiation, the waiting time between successive initiation events is gamma distributed [55]. As a result the distribution of nascent RNAs is expected to be narrower than Poisson with a Fano factor less than one. The presence of transcriptionally inactive states on the other hand has the effect of broadening the distribution of nascent RNAs, and should lead to a Fano factor greater than one in the case when initiation from the active state is a one-step process.

For bursty promoters that switch between an active and an inactive state (for example the PDR5 gene in yeast [4]) the nascent RNA distribution can also be used to discriminate between different mechanisms of regulation. Recent experiments [57,58] have suggested that transcriptional regulation may be achieved by either modulation of the burst size (given by *k*_*INI*_/*k*_*OFF*_, where *k*_*INI*_ = *k*_*LOAD*_ *× k*_*ESC*_ */* (*k*_*LOAD*_ *+ k*_*ESC*_) is the average rate of initiation), or by modulating the burst frequency (*k*_*ON*_); it is also possible that both are tuned [7]. In Fig 2C we show the results of our calculations of the Fano factor for the nascent RNA distribution, using parameters that are characteristic of the PDR5 gene and assuming that transcriptional regulation is achieved either by tuning the burst size or the burst frequency. We see that even though both mechanisms of regulation produce Fano factors larger than one, they make qualitatively different predictions for the functional dependence of the Fano factor on the mean number of nascent RNAs.

### Mean and variance of the nascent RNA distribution in the large gene-length limit

Nascent RNA distribution is determined by stochastic initiation and elongation. However for a long gene, the elongation process becomes practically deterministic due to the law of large numbers. Assuming as we do in our model that each elongation step is a stochastic process, with the same rate *k*,the elongation time will be Gaussian distributed with a mean and variance that are proportional to the length of the gene. Therefore the deviation of the elongation time away from the mean compared to the mean will decrease as the square root of the gene length. A recently published paper by Senecal et al.[37] has explored how stochastic initiation and deterministic elongation processes affect nascent RNA distribution.

**Fig 3:**
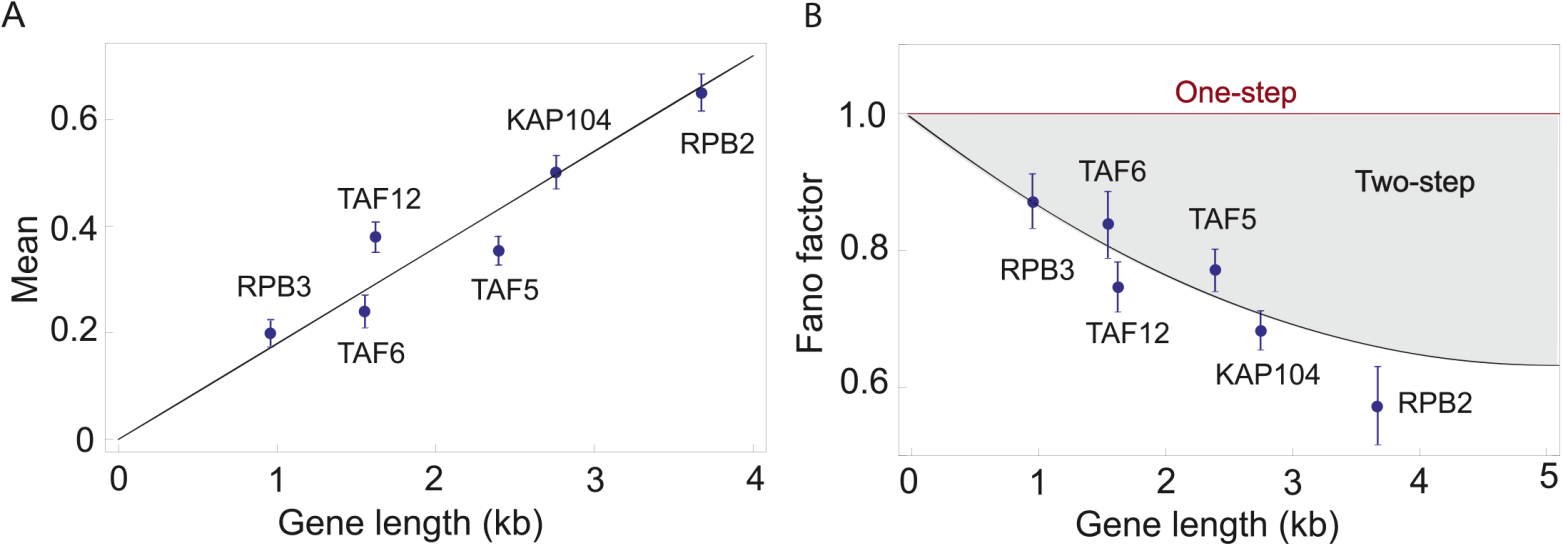
(A) Mean number of nascent RNAs for six different yeast genes. We use the nascent RNA distribution data for six constitutively expressed yeast genes: KAP104, TAF5, TAF6, TAF12, RPB2, RPB3 and plot the mean of the distributions as a function of the gene length. The mean of the distribution increases linearly with the gene length indicating that the transcription of all six genes is initiating at the same rate. The initiation rate of these genes extracted from the data is 0.145±0.025/min, where the rate of elongation is taken to be 0.8 kb/min [4]. **(B) Fano factor for the nascent RNA distribution of six different yeast genes.** Using the data for the nascent RNA distributions for the same six yeast genes described in (A) we compute the Fano factor and compare it to predictions from our model. The shaded region shows the possible values that the Fano factor can take depending on the ratio of *k*_*LOAD*_ and *k*_*ESC*_ given the initiation rate determined from the mean in part (A). The boundary of the shaded region corresponds to the minimum amount of noise (as measured by the Fano factor) given the extracted rate of initiation in part (A), and it is obtained when the two rates are the same, i.e., *k*_*LOAD*_ = *k*_*ESC*_ = 0.29±0.013/min. Interestingly enough the Fano factors characterizing the nascent RNA distribution for these six yeast genes seem to lie on this boundary. (The nascent RNA data for the six yeast genes used in our analysis is taken from ref. [25].)

However, and although this is indeed the case for FISH data such as the one analyzed in Fig 3, in the paper, an important application of our method will be the analysis of single-cell sequencing data, where the positions of every polymerase along the gene can be determined. In addition, and as shown in Fig 1, we anticipate that our method can be applied to electron micrograph data (e.g. such as those reported in [29,31]), a method that also allows one to measure the position of each polymerase along a transcribed gene. Using this technique, the number of polymerases in the first *L* nucleotides of the gene can also be determined, and statistics (mean and variance) can be computed and compared to experimental results. This will allow us to computationally bin a gene into smaller chunks of arbitrary length, and use length as a “data analysis” turning knob that we can tune computationally to investigate how it affects the noise(Fano factor). To be able to do this, a fluctuating elongation rate is essential, since in principle the gene length can be made as short as desired during data analysis.

In the limit of a long gene, when the residence time of the RNAP on the gene is practically deterministic, we can use queuing theory to compute closed form expressions for the first and second moments of the nascent RNA distribution [59,60]. For the one-step model, the promoter is always active (*k*_*OFF*_ → 0 in Fig 2A) and there is a single rate-limiting step leading up to initiation. In this case the nascent RNA distribution is characterized by a variance that is equal to the mean, which is what we computed for stochastic elongation as well. The second limit of interest is when the rates of assembly of the transcriptional machinery (*k*_*LOAD*_) and promoter escape (*k*_*ESC*_) have comparable magnitudes, i.e., transcription initiation is a two-step process. For an elongation time *T = L/k* (where *L* is the number of bases along a gene and *k* is the average rate of elongation) the mean and variance are given by

$$\begin{array}{c} \left\langle M\right\rangle =\frac{k_{LOAD}k_{ESC}T}{k_{LOAD}+k_{ESC}}\\ Variance=\left\langle M\right\rangle \left[1-\frac{k_{ESC}k_{LOAD}T}{\left(k_{ESC}+k_{LOAD}\right)}+\frac{k_{ESC}k_{LOAD}}{T{\left(k_{ESC}+k_{LOAD}\right)}^{2}}[2(\frac{1-\exp(-\left(k_{ESC}+k_{LOAD}\right)T)}{\left(k_{ESC}+k_{LOAD}\right)})-2T+T^{2}(k_{ESC}+k_{LOAD})]\right]. \end{array}$$

The Fano factor can hence be computed very easily by taking the ratio of the variance and mean. It is to be noted that when one of the rates that describe the two steps leading to initiation (*k*_*LOAD*_ and *k*_*ESC*_) becomes much smaller than the other, we are back to the case of one rate-limiting step. In the case of one rate-limiting step the Fano factor becomes one, which is the signature of a Poisson initiation process. However when the rates *k*_*LOAD*_ and *k*_*ESC*_ become comparable, the Fano factor is reduced from 1 and attains a minimum value when *k*_*LOAD*_ is equal to *k*_*ESC*_. This also follows from the equations for the variance and the mean and can be intuited by noting that the two rates appear in the equations in a symmetrical fashion.

The third limit of interest is the ON-OFF model of initiation which is characterized by *k*_*OFF*_ (the rate of promoter switching from the ON to OFF state), *k*_*ON*_ (the rate of promoter switching from the OFF to ON state), *k*_*ESC*_ (the rate of escape, which we assume is much higher than the rate of assembly of the transcriptional machinery), and time of elongation *T*. The mean and variance are given respectively by,

$$\begin{array}{c} \left\langle M\right\rangle =\frac{k_{ON}k_{ESC}T}{k_{ON}+k_{OFF}}\\ Variance=\left\langle M\right\rangle \left[1+\frac{2k_{ESC}k_{OFF}}{{\left(k_{ON}+k_{OFF}\right)}^{2}}+\frac{2k_{ESC}k_{OFF}}{{\left(k_{ON}+k_{OFF}\right)}^{3}}(\frac{\exp(-\left(k_{ON}+k_{OFF}\right)T)-1}{T})\right]. \end{array}$$

As shown in S4 Fig, these formulas give almost identical results to those we obtain when taking into account stochastic elongation, when the gene length is of the order of few thousand bases.

### Nascent RNA distributions in yeast are consistent with a two-step mechanism of transcription initiation

The theoretical results described above can be used as a mathematical tool to extract information about transcription initiation dynamics from nascent RNA distributions, which have been measured in a series of recent experiments [4,25]. To demonstrate the utility of this approach, we analyze a set of nascent RNA distributions for twelve different constitutively expressed genes in yeast [25]. We find that for six of these twelve genes (RPB2, RPB3, TAF5, TAF6, TAF12, KAP104), the mean number of nascent RNAs scales linearly with the gene length, as shown in Fig 3A. If we assume that all of these genes have comparable elongation rates (*k* = 0.8 kb/min (4)), then the linear relationship between the mean nascent RNA number and gene length implies that the average initiation rates of these genes are all roughly the same and equal to *k*_*INI*_ = 0.145±0.025/min.

In addition to the mean, our model allows us to investigate the behavior of the variance of the nascent mRNA distribution with gene length, and compare it to the predictions from different models of transcription initiation. Given that the Fano factors of the nascent RNA distribution for the six genes, RPB2, RPB3, TAF5, TAF6, TAF12, and KAP104, are all less than one, the simplest model consistent with the data is one where the promoter is always active and transcription initiation is a two-step process (see Fig 2A).

This model is parameterized by the rates *k*_*LOAD*_ and *k*_*ESC*_. The Fano factor of the nascent RNA distribution depends on the ratio of these two rates. Our model makes prediction for how the Fano factor changes with the gene length when the rates *k*_*LOAD*_ and *k*_*ESC*_ are tuned, consistent with the mean initiation rate being *k*_*INI*_ = *k*_*LOAD*_ *× k*_*ESC*_ */* (*k*_*LOAD*_ *+ k*_*ESC*_) = 0.145±0.025/min. As shown in Fig 3B, this value of the initiation rate defines a region in the Fano factor–Gene length phase (light-blue shaded area in Fig 3B). This region is bounded on its upper side by the limit when one of the rates (either *k*_*LOAD*_ or *k*_*ESC*_) is much larger than the other one (which turns initiation into a one-step process with a Fano factor equal to one), and on the lower side, by the limit in which the two rates are identical. The limit of identical rates gives the minimum Fano factor attainable when the average initiation rate is 0.145±0.025/min. Remarkably, we find that the six genes in question have the lowest possible Fano factor. In principle, the six genes shown in Fig 3A could have ended up anywhere within the shaded region in Fig 3B. The fact that they all follow the lower boundary of the allowed region suggests that these genes, which have varying length, have not only the same average initiation rate, but also that they have identical promoter cycling kinetics, with roughly the same values of *k*_*LOAD*_ and *k*_*ESC*_ (*k*_*LOAD*_ = *k*_*ESC*_ = 0.29±0.013/min).

It is to be noted that a multi-step initiation model, where initiation happens in more than two sequential steps (this can also include bursting kinetics) can also account for the Fano factor for the six different genes being less than one. However a more complicated model with more than two sequential steps will have many free parameters e.g. for a three step model we will have three sequential steps to initiation characterized by three different rates. Although we cannot rule out such possibilities, the two-step model in spite being the simplest possible scenario explains the data well and provides mechanistic insight into the dynamics of initiation for the six different genes. The key result here is that by analyzing nascent RNA distributions, we can exclude the one-step and ON-OFF models of initiation.

The remaining six (RPB1, MDN1, PUP1, PRE7, PRE3, PRP8) constitutive genes of the twelve studied [25] initiate at rates that are different than the rate of initiation that we found for the six genes discussed above (see S2 Fig). All but one of these six genes have nascent RNA Fano factors that are less than one, consistent with two or more steps leading up to initiation. This second set of genes thus acts as a control group that, as expected for a set of genes having different gene-specific rates of transcription, occupies the allowed region in the Fano factor-Gene length phase space without clustering at the lower boundary of this region, like we found for the six genes discussed above (S3 Fig).

## Discussion

Direct imaging of transcriptional dynamics in real time [19–23] at the molecular scale and in individual cells still remains challenging. As an alternative, a number of recent studies have tried to decipher the dynamics of transcription initiation using the measured cell-to-cell variability of transcriptional outputs (cytoplasmic messenger RNA or protein molecules) at the single cell level [1,3,4,25,61]. These measurements of transcriptional cell-to-cell variability have been interpreted in the context of a classification scheme for promoters, which are characterized by either a Poisson or a Gamma distribution of their outputs. These differences have then been taken to indicate a difference in the mechanism of transcription. A Poisson distribution is taken as evidence that the promoter transcribes at a constant rate, i.e., initiation is a one-step process. The Gamma distribution on the other hand is indicative of bursty promoter dynamics [4,19]. In practice, the distribution of cytoplasmic mRNA or proteins obtained from a population of cells is fitted to a mathematical model that incorporates the stochastic kinetics of transcription (and translation in the case of proteins), and the fitting parameters are interpreted as representative of the kinetic properties of stochastic gene expression (e.g., burst size, burst frequency, average transcription rate, etc.) [5]. Even though in some cases this approach has produced kinetic parameters whose values are consistent with direct measurements of the same parameters [1], the interpretation of the kinetic parameters can be difficult given that the distributions of mRNA and protein may be affected by stochastic processes that occur downstream of transcription initiation. Examples of these processes include the non-linear degradation of mRNA and proteins [14], maturation time of fluorescent reporters [62], transport of mRNA out of the nucleus [10,11], mRNA splicing [12,13] and small RNA regulation [14,63]. Furthermore, recent theoretical results [15,16] indicate that fluctuations due to random partitioning of molecules during cell division may yield the same mathematical dependence between variance and mean of protein and mRNA copy number in clonal cell populations, as would a stochastic model of transcription initiation and linear degradation.

**Fig 4:**
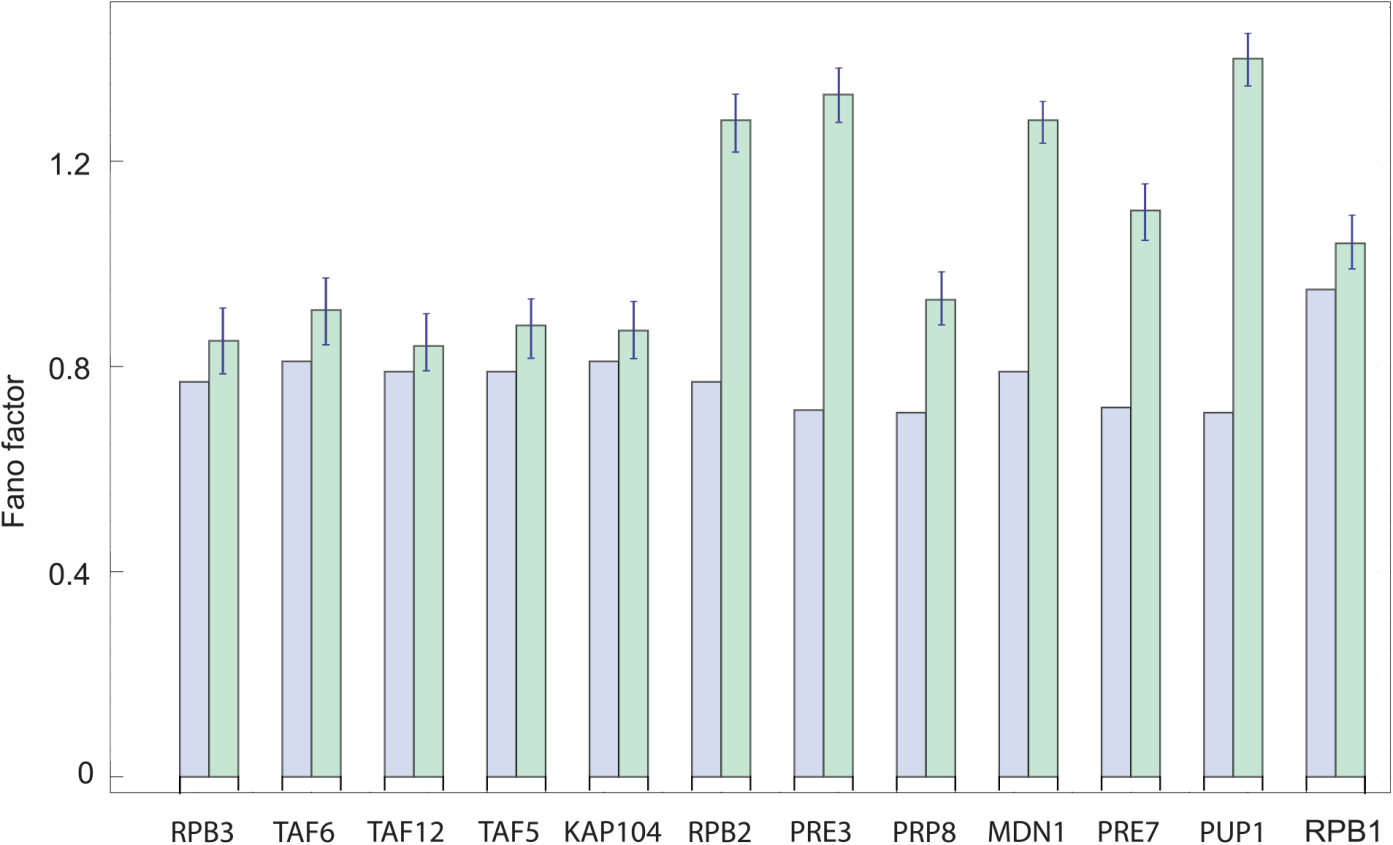
Comparison of predicted and measured Fano factors for cytoplasmic mRNA distributions. Fano factors for the cytoplasmic mRNA distributions, as predicted by the one-step (RPB1), two-step (KAP104, TAF5, TAF6, TAF12, RPB2, RPB3, MDN1) and three-step (PUP1, PRE3, PRE7, PRP8) mechanisms of initiation, are shown as blue bars. These are compared with the measured cytoplasmic mRNA distributions, shown in green bars, as reported in ref [25]. In cases when the measured distributions have higher Fano factors than predicted, this is indicative of significant sources of noise downstream to transcription initiation and elongation that affect the cell-to-cell variability of cytoplasmic mRNA.

In order to demonstrate that the distribution of mRNAs can be affected by stochastic processes that occur downstream of transcription, thereby obscuring the signature of transcription initiation dynamics, we compare the nascent RNA and cytoplasmic mRNA distributions for the twelve yeast genes analyzed in Fig 4. First, we compute the Fano factor of the cytoplasmic mRNA distribution predicted by the initiation mechanism inferred from the measured nascent RNA distribution for all twelve genes studied (23). (See the S1 Text for details of the calculation.) Then we compare the results of our calculations with the experimentally determined distributions obtained by counting cytoplasmic mRNA. We find that for all of the yeast genes examined the predicted Fano factors for the cytoplasmic mRNA distributions are less than the measured ones, as shown in Fig 4. In other words the signature of two-step initiation observed in the nascent RNA distribution is washed out at the cytoplasmic mRNA level due to other sources of noise. It remains unclear what processes are responsible for these differences. In a recent study of transcription in fly embryos, it was also found that the variability of nascent and cytoplasmic mRNA could differ more than six fold [9]. In this case, the reason for this difference is spatial and temporal averaging of mRNA by diffusion and accumulation of mRNA transcripts during nuclear cycles. The yeast and fly examples demonstrate that the relationship between nascent and cytoplasmic RNA distributions is complex and context dependent.

An alternative to counting cytoplasmic proteins or mRNA is to count the number of transcribing polymerases [27–30], or nascent RNAs [4,25,37] on the gene being transcribed, using electron micrographs and fluorescence in situ hybridization, respectively. These measurements are not affected by post-transcriptional processes and are more direct readouts of transcriptional dynamics. To date, these distributions of nascent RNAs have been used mostly in a qualitative manner, due to the lack of mathematical models that connect these distributions with the underlying mechanisms of transcription, apart from the recent paper by Senecal et al. [37]. For instance, distributions of nascent RNAs (or of transcribing RNA polymerases) have been recently reported in yeast [4,23,25], fly embryos [9,49,64], and bacteria [29,65]. The model of transcription initiation and elongation developed here offers a way to quantitatively analyze these measured nascent RNA distributions, and connect them to molecular mechanisms of transcription. In particular, when we consider three different models of transcription initiation that incorporate three broad classes of initiation mechanisms, we find that they make qualitatively different predictions for nascent RNA distributions.

Analyzing the nascent RNA distributions for twelve constitutively expressed genes in yeast [25], we find that all but one of these distributions have a Fano factor less than one. This observation is consistent with a simple model in which initiation proceeds in two-steps (for some of the genes more than two steps are implicated by the data; see S3 Fig), which are of similar duration. The two rate limiting steps can arise from a number of different sources. For the genes in yeast considered in the paper the initiation complex is formed by assembly of multiple transcription factors and co-factors [66]. After the formation of the initiation complex, an RNAP molecule initiates transcription by escaping the promoter. The two-step model we consider in the paper would be realized if out of all these steps leading up to initiation any two steps become rate-limiting. The most surprising finding when analyzing these twelve genes was that six of them have not only the same average initiation rate, but also the same rates of loading of the transcriptional machinery, and of promoter escape. We do not have a mechanistic interpretation for this finding, but the data suggests the existence of a common molecular mechanism of initiation for these six genes, and given that they represent half of the genes in the data set we have analyzed here, it is tempting to speculate that other yeast genes may share the same kinetics. More experiments are clearly needed to test this hypothesis, ideally ones where the dynamics of transcription are followed directly [23].

Our findings for the yeast promoters, highlight the utility of our theory for deciphering transcriptional dynamics in vivo from nascent RNA distributions. In addition, counting nascent RNAs, mRNAs and proteins simultaneously will undoubtedly further enhance our understanding of how the central dogma of molecular biology plays out in individual cells.

## Materials and Methods

### Calculation of moments of the nascent RNA distribution

To compute the first two moments of the nascent RNA distribution for the canonical model of transcriptional regulation shown in Fig 2A we apply the general method of deriving moment equations from the master equation, Eq (2). The rate matrices that define the master equation, Eq (2), are in this case:

$$\begin{array}{l} \hat{K}=\left[\begin{array}{ccc} -k_{ON} & k_{OFF} & 0\\ k_{ON} & -(k_{OFF}+k_{LOAD}) & k_{ESC}\\ 0 & k_{LOAD} & -k_{ESC} \end{array}\right],\\ \hat{R}=\left[\begin{array}{ccc} 0 & 0 & 0\\ 0 & 0 & k_{ESC}\\ 0 & 0 & 0 \end{array}\right],\\ \hat{\Gamma }=\left[\begin{array}{ccc} k & 0 & 0\\ 0 & k & 0\\ 0 & 0 & k \end{array}\right]. \end{array}$$

Here $\hat{K}$, is the transition matrix, which describes promoter switching between the three possible states shown in Fig 2A. When an RNA polymerase initiates transcription from the state in which the polymerase is bound to the promoter, the state of the promoter changes to the state in which the promoter does not have a bound polymerase. This accounts for the rate of escape appearing in the transition matrix and also explains why $\hat{R}$ (the initiation rate matrix) is not diagonal. Using these matrices in the master equation for the nascent RNA distribution (Eq (1)) we compute analytically the mean and the variance of the distribution as a function of the gene length *L*. These results were used to make the plots in Fig 2B and 2C.

### Limitations of the model

Our model (S1B Fig) makes the assumptions that RNAP molecules do not pause and do not collide with other RNAP molecules, while moving along the gene. We also take the size of the RNAP footprint to be one base, and we do not restrict the number of RNAPs at each base along the gene. These assumptions are equivalent to the assumption that the average number of transcribing RNA polymerases is much less than one per base. If we consider a constitutive (one-step) promoter with an initiation rate *k*_*ESC*_, and the rate at which the RNAP translocates from one base to the next is *k*, then the number *m* of RNAP molecules on the first base of the gene would be Poisson distributed [40] and given by,

$$P(m)=\frac{{\left(\frac{k_{ESC}}{k}\right)}^{m}}{m!}e^{-\frac{k_{ESC}}{k}}.$$

As the above equation demonstrates, if the ratio of initiation rate and hopping rate *k*_*ESC*_/*k* is of the order of 0.01 (characteristic of MDN1 promoter [25)], the probability of finding two or more RNAP molecules at the first base of the gene would be 5×10^−5^. This justifies one of the main assumptions of our model, namely that we can ignore the constraint that no base can be occupied by more than one polymerase. The assumption will be valid as long as the initiation time scale is slower than the elongation time scale, and it makes the model analytically tractable. As described in the results section, this assumption leads to simple formulas for the first two moments of the nascent RNA distribution in the large gene-length limit, and to a set of *L* ^*2*^ linear equations in the case of stochastic elongation. As shown in the S1 Text, these linear equations are readily solved to obtain the moments of the nascent RNA distribution using standard computing tools such as Mathematica or Matlab. This is important in order to test many parameter sets without having to run a new Gillespie simulation for every set which can be impractical for complex kinetic mechanisms of transcription initiation.

As argued above, we expect the approximations made in our model to be reasonable for all but the strongest promoters characterized by very fast initiation [43,44].

In order to test this intuition, we compare the analytic predictions of our model with numerical simulations of a more realistic one (referred to as the traffic model in S1A Fig), which properly accounts for the footprint of a transcribing RNAP molecule on the DNA, ubiquitous pausing of the polymerase, and excluded volume interactions between adjacent polymerases along the gene. In particular we compare the mean and the Fano factor of nascent RNA distributions, as predicted by our model of transcription for the case when initiation occurs via a single rate limiting step, with those obtained from numerical simulation of the traffic model obtained using the Gillespie algorithm [45,46].

A single time step of the simulation is performed in the following way: one of the set of all possible reactions is chosen at random according to its relative weight, which is proportional to the rate of the reaction, and the state of the system is updated by implementing the change described by the chosen reaction. The time elapsed since the last step is drawn from an exponential distribution, the rate parameter of which equals the sum of all the rates of the possible reactions at that time. This process is repeated for a long enough time such that the number of RNAP molecules along the gene (which is the same as the number of nascent RNAs) reaches steady state.

We consider four different transcription initiation rates, spanning the typically observed values in *E*. *coli* and yeast cells [4,19,23,25], and we observe in the simulations how the mean and Fano factor of the nascent RNA distribution are affected by RNAP pausing and road blocking (S1A and S1B Fig). We find that for initiation rates slower than 30 initiations/min, both the mean and the Fano factor extracted from the simulations are in good agreement (less than 10% difference) with the analytical results (S1C and S1D Fig). In simulations we used the following parameters to describe RNAP elongation: *k*_*P-*_ *=* 4/sec, *k*_*P+*_ *=* 0.01/sec, *k =* 80 bp/sec, as was reported for ribosomal promoters in *E*.*Coli* [43]. We also use a gene of length *L* = 2000 bases and a polymerase whose DNA footprint is 30 bases.

### Parameter selection

We generated the plots for the Fano factor versus gene length (Fig 2B), for the three limits of the model in Fig 2A using the parameters listed below. For the bursty promoter, where the promoter slowly switches between inactive and inactive states, we use *k*_*OFF*_ = 5/min, *k*_*ON*_ = 0.435/min, *k* = 0.8kb/min, *k*_*LOAD*_ *=* 5/min and *k*_*ESC*_ *=* 0/min; *k*_*OFF*_, *k*_*ON*_, *k*, and *k*_*LOAD*_ are the characteristic rates for the PDR5 promoter, as reported in [4]. For the two-step initiation model, where the promoter does not switch between an active and an inactive state but has two rate limiting steps leading up to initiation, we use *k*_*LOAD*_ *=* 0.14/min, *k*_*ESC*_ *=* 0.14/min, *k*_*OFF*_ = 0/min, *k*_*ON*_ = 0/min, *k* = 0.8kb/min; these are characteristics of yeast genes, such as MDN1 [25]. For the one-step model, there is one rate limiting step leading up to transcription elongation and we choose *k*_*LOAD*_ *=* 0.09/min, *k*_*ESC*_ *=* 0/min, *k*_*OFF*_ = 0/min, *k*_*ON*_ = 0/min, *k* = 0.8kb/min, which are characteristics of the yeast gene RPB1 [25].

Genes that are transcribed from a promoter that switches between an active and an inactive state can be regulated by changing the rates of switching between these two states, either by modulating the burst size (given by *k*_*INI*_/*k*_*OFF*_, where *k*_*INI*_ = *k*_*LOAD*_ *k*_*ESC*_ */* (*k*_*LOAD*_ *+ k*_*ESC*_) is the average rate of initiation), or by modulating the burst frequency (*k*_*ON*_), (it is also possible that both are modulated) [57,58]. In order to compute the predictions for the nascent RNA distribution for these two mechanisms of regulation in Fig 2C, we change burst size and burst frequency by changing *k*_*OFF*_ and *k*_*ON*_. In the first case, we change the burst size by changing *k*_*OFF*_ and taking the other parameter values to be, *k*_*ON*_ = 0.435/min, *k* = 0.8kb/min, *L* = 4436 bps, *k*_*INI*_ *=* 5/min as reported for PDR5[4]. Then we change burst frequency by changing *k*_*ON*_, where the other parameters are, *k*_*OFF*_ = 5/min, *k* = 0.8kb/min, *L* = 4436 bps, *k*_*INI*_ *=* 5/min as reported for PDR5 [4]. In Fig 2C the Fano factor of the nascent RNA distribution is plotted as a function of its mean normalized by mean_max_, where mean_max_ is the maximum of the mean number of nascent RNAs which is obtained when there is no transcriptional regulation and the promoter is always active.

### Data analysis: Yeast genes

We analyze the measured nascent RNA distributions for twelve different constitutively expressed yeast genes reported in reference [25]. By applying our theoretical results to the published data, we find that the average initiation rates of six (KAP104, TAF5, TAF6, TAF12, RPB2, RPB3) of these twelve genes are all roughly the same, and equal to 0.145±0.025/min. However the other six genes (RPB1, MDN1, PUP1, PRE7, PRE3, PRP8) initiate transcription at different rates. This we conclude from S2 Fig, where the mean number of nascent RNAs is plotted against the gene length for all the twelve genes.

When considering experiments that count nascent RNAPs it is important to be mindful of the fact that the number of RNAP molecules along a gene is not necessarily equal to the nascent RNA counts. Transcribing RNAPs have partial nascent transcripts attached to them depending on how far along the gene they have progressed (as indicated in Fig 1C). In a single molecule FISH experiment, the RNA sequence that is targeted by the fluorescent probes determines if these transcripts are detected or not. Probes against the 5’ end detect transcripts early on, while probes against the 3’ end will detect only almost finished transcripts. [9]. However, as long as there is a way to correctly extract the RNAP number distribution from nascent RNA intensity, our model can accurately transform this data into information about the transcriptional dynamics.

In addition to the mean, we analyze the Fano factor of the nascent mRNA distributions as well, and compare it with the prediction from our model of transcriptional regulation (Fig 2A). It is to be noted that for 9 of the different genes we consider, the number of nascent RNAs does not exceed 2. These distributions can be described by the probabilities of having 0,1 and 2 nascent RNAs. Still the Fano factor is a useful metric for analyzing these distributions and quantifying how much they differ from a Poisson distribution. We find the Fano factors of the nascent mRNA distribution for all of the twelve genes in the data set to be less than (or at most equal to) one. Hence the simplest model consistent with the published data for these twelve yeast genes is one where the promoter is always active and transcription initiation is a two-step process (see Fig 1A) parameterized by the kinetic rates *k*_*LOAD*_ and *k*_*ESC*_. We find that Fano factors for the six genes: KAP104, TAF5, TAF6, TAF12, RPB2, RPB3, which all initiate transcription at the same average rate, follow precisely the trend-line expected when *k*_*LOAD*_ and *k*_*ESC*_ are equal (*k*_*LOAD*_ = *k*_*ESC*_ = 0.29±0.013/min), as shown in Fig 2B.

We also analyze the Fano factor of the nascent mRNA distributions of the other six genes: RPB1, MDN1, PUP1, PRE7, PRE3, PRP8, which initiate transcription at a different mean rate (S2 Fig). Their location in the phase space defined by the gene length and Fano factor (as shown in S3 Fig) indicates that they all have at least two rate limiting steps leading up to initiation and that these steps are likely parameterized by different rates, unlike what we observe for the other six genes.

It is to be noted that it might be difficult to experimentally tell the difference between a mature RNA or a single nascent RNA, there are 12 different genes for which data is available [25] and our conclusions are based on examining the whole set. As pointed out earlier 9 of these genes have up to 2 nascent RNA molecules. Hence for these genes the distributions of nascent RNA molecules have three bins (for 0,1 and 2 mRNA molecules respectively). However our analysis also includes genes for which there are more than 1 or 2 nascent RNAs, such as RPB1 (up to 3), MDN1 (up to 5), PRP8 (up to 3). We find all of these genes to have a Fano factor of less than one indicative of two or more steps leading to initiation.

### Comparison of the cytoplasmic and nascent RNA distributions in yeast

In order to compare the nascent RNA and cytoplasmic mRNA distributions, we compute the Fano factor of the cytoplasmic mRNA distribution, predicted by the two-step mechanism of initiation for the seven genes (KAP104, TAF5, TAF6, TAF12, RPB2, RPB3, MDN1). In other words an mRNA molecule is produced in two sequential steps, e.g., by first assembling the transcriptional machinery at the promoter DNA, followed by RNA polymerase escaping the promoter. These two steps are parameterized by the kinetic rates *k*_*LOAD*_ and *k*_*ESC*_, respectively. For the gene RPB1 initiation is a one-step process, while for others (PUP1, PRE3, PRE7, PRP8) three steps are required to account for the measured nascent RNA distribution. We further assume that mRNA is degraded with a constant probability γ per unit time per molecule. The degradation rates of the twelve genes used in the calculation are those reported in reference [25]. Given these assumptions about mRNA production and degradation, we compute the Fano factor (ratio of variance and mean) of the mRNA distribution using the approach developed previously in order to find the moments of mRNA distribution [8,38–41]. The computed Fano factor is a measure of the expected cell-to-cell variability in the number of cytoplasmic mRNAs *if* only the initiation process was contributing to this variability. The fact that we observe a discrepancy between the mRNA variability calculated in this way and the measured mRNA variability (Fig 4) is indicative of the presence of significant sources of noise that are downstream of transcription.

## Supporting Information

S1 Text Calculation of the first two moments of the nascent RNA distribution.
(DOCX)

S1 Fig (A) Two state promoter with traffic. The promoter switches between two states: state *1*, from which transcription initiation occurs with a constant probability per unit time *k*_*1*,*ini*_, and state 2, from which transcription does not initiate. The promoter switches from state 1 to state 2 with probability per unit time *k*_*12*_, and from 2 to 1 with probability per unit time *k*_*21*_. After initiation each RNAP molecule hops from one base pair to the next along the gene at a rate *k* per unit time. Each RNAP molecule has a finite DNA footprint of *30 bp* and it can pause at any site with a rate *k*_*P+*_ and come out of the pause with a rate *k*_*P-*_. An RNAP molecule cannot move forward if another one occupies the bases in front of it. The length of the gene is *L*. **(B) No-traffic model.** The elongation process is uniform with each RNAP molecule occupying one base pair. **(C)** Using the Gillespie algorithm [45,46], we simulate the traffic model where the promoter initiates at a constant rate, where we take four different values of initiation. Predictions of the no-traffic model for the mean and Fano factor agree well with the simulation results from the traffic model up to an initiation rate of 30 initiations/min. The elongation parameters used, are *k*_*P-*_ *=* 4/sec, *k*_*P+*_ *=* 0.01/sec, *k =* 80 bp/sec [43] as reported for ribosomal promoters in *E*.*Coli*. The length of the gene is *L* = 2000 bps and the footprint of one RNAP molecule is 30 bps.
(EPS)

S2 Fig Mean number of nascent RNAs in yeast. Here we plot the mean of the nascent RNA distribution for all twelve genes: MDN1, PRP8, RPB1, PUP1, PRE3, PRE7, KAP104, TAF5, TAF6, TAF12, RPB2, RPB3, reported by Gandhi et al. [25]. For six of these twelve genes, KAP104, TAF5, TAF6, TAF12, RPB2, RPB3, the mean increases linearly with the gene length, as shown in the main text in Fig 2A. The other six genes do not follow the same trend line indicating that they have different initiation rates; PUP1, PRE3, PRE7, have similar initiation rates but different from the six genes analyzed in the main text.
(EPS)

S3 Fig Fano factor for nascent RNA distributions in yeast. Here we show 6 different constitutively expressed genes in yeast [25]: RPB1, MDN1, PUP1, PRE7, PRE3, PRP8 which have different initiation rates than the six genes shown in Fig 2, and another gene PDR5, known to be regulated [4]. The data for Fano factors measured in experiments are shown in comparison with the predictions for the one-step (red line) and two-step (area between red line and blue line) initiation models. The Fano factor for the PDR5 gene is greater than one, which is consistent with an ON-OFF model of transcription initiation. RPB1 has a Fano factor equal to 1, suggesting one-step initiation. All the other genes have Fano factors consistent with more than one rate-limiting step leading up to initiation. The blue line indicates the minimum possible Fano factor for the specific gene assuming two-step initiation and the measured average initiation rate, which we compute from the mean number of nascent RNAs. For the genes whose Fano factor is below this line (PUP1, PRE7, PRE3, PRP8) the data suggest that their transcription is initiated via three or more (similar in duration) steps.
(EPS)

S4 Fig Comparison between stochastic and deterministic elongation. We compare the Fano factors for the one step, two-step and ON-OFF models with stochastic and deterministic elongation respectively, when the gene length is tuned. When the gene length is of the order of few thousand bases, the elongation process essentially becomes deterministic and hence the Fano factors for the models become similar for all the initiation models considered. To illustrate this point for the ON-OFF model we use the following parameters: *k*_*OFF*_ = 5/min, *k*_*ON*_ = 0.435/min, *k* = 0.8kb/min, *k*_*LOAD*_ *=* 5/min and *k*_*ESC*_ *=* 0/min, which are characteristic of the PDR5 promoter in yeast, as reported in [4]. For the two-step model we use *k*_*LOAD*_ *=* 0.14/min, *k*_*ESC*_ *=* 0.14/min, *k*_*OFF*_ = 0/min, *k*_*ON*_ = 0/min, *k* = 0.8kb/min, characteristic of MDN1 promoter, which we find by analyzing the data reported in ref. [25]. For the one-step model, we use *k*_*LOAD*_ *=* 0.09/min, *k*_*ESC*_ *=* 0/min, *k*_*OFF*_ = 0/min, *k*_*ON*_ = 0/min, *k* = 0.8kb/min, which are characteristics of the yeast gene RPB1, obtained by analyzing the data published in ref. [25].
(EPS)
