# Supplementary material for: Deciphering Transcriptional Dynamics In Vivo by Counting Nascent RNA Molecules
Source: PLoS Comput Biol. 2015 Nov 6;11(11):e1004345. doi: 10.1371/journal.pcbi.1004345 (PMC4636183; doi:10.1371/journal.pcbi.1004345)
Supplement: S1 Text — Calculation of the first two moments of the nascent RNA distribution. (DOCX) [file pcbi.1004345.s001.docx]

**S1 Text. First two moments of the nascent RNA distribution**

In order to connect mechanisms of transcription initiation with nascent RNA distributions, in the results section we described a model of transcriptional dynamics with an arbitrarily complex initiation mechanism followed by an elongation process. We make the following assumptions in our model (Supplementary Figure 1B): after initiation, each RNA polymerase molecule elongates along the gene by hopping from one to the next base at a constant probability per unit time. RNAP molecules do not pause and nor do they collide with each other, while moving along the gene. We also take the size of the RNAP footprint to be one base, and we do not restrict the number of RNAPs at each base along the gene. Here we present details of the calculations leading up to analytic formulas for the first two moments of the nascent RNA distribution predicted by this model.

To compute the first two moments of the nascent RNA distribution for arbitrary promoter architecture, we consider a promoter that can exist in *N* possible states. The rate of going from the *s-th* to the *q-th* promoter state is *k_s,q_*, and the rate at which an RNAP molecule initiates transcription from the *s*-*th* promoter state is *k_s,ini_*. After initiation every RNAP molecule elongates by hopping from one base to the next at a constant rate *k* and the number of nascent RNAs at site *i* is *m_i_*. If the number of bases along the gene is *L,* then the number of nascent RNAs along the gene will be given by

$M=\sum_{i=1}^{L} m_{i}.$ (3)

Here *m_i_*’s are dependent random variables since at each base the number of nascent RNAs (*m_i_*) depends on the number of nascent RNAs present on the previous base (*m_i-_*_1_). The state of the gene is described by (*L* + 1) stochastic variables: the number of nascent RNAs (*m_1,…_,m_L_*) at each base along the gene, and the state of the promoter, *s*. Thus, the probability distribution function describing the gene is given by *P*(*s*, *m_1,…_,m_L_* ). The time evolution for this probability can be calculated using the chemical master equation approach:

$\begin{matrix} \frac{dP(s,m_{1},...,m_{L})}{dt}=k_{s,ini} P(s,m_{1}-1,...,m_{L})+\sum_{i=1}^{L} k (m_{i}+1) P(s,m_{1},.,m_{i}+1,m_{i+1}-1,.,m_{L}) \\ -(k_{s,ini}+\sum_{i=1}^{L} k m_{i}+\sum_{q} k_{s,q}) P(s,m_{1},.,\mathrm{m}_{i},.,m_{L})+\sum_{q} k_{q,s} P(s,m_{1},...,m_{L}) . \end{matrix}$ (4)

Similar approaches have been used previously in order to find the moments of mRNA and protein distributions[1–4]

. By defining the probability vector$\vec{P}(m_{1},...,m_{L})=(P(1,m_{1},...,m_{L}),P(2,m_{1},...,m_{L}),...,P(s,m_{1},...,m_{L}))$, we can rewrite equation (4) in compact matrix form as

$\begin{matrix} \frac{d\vec{P}(m_{1},...,m_{L})}{dt}=(\hat{K}-\hat{R}-\hat{\Gamma}\sum_{i=1}^{L} m_{i}) \vec{P}(m_{1},.,\mathrm{m}_{i},.,m_{L})+\hat{R} \vec{P}(m_{1}-1,...,m_{L}) \\ +\sum_{i=1}^{L-1} k (m_{i}+1) \hat{\Gamma} \vec{P}(m_{1},.,m_{i}+1,m_{i+1}-1,.,m_{L}) + k(m_{L}+1) \hat{\Gamma} \vec{P}(m_{1},...,m_{L}+1). \end{matrix}$ (5)

Here we introduce the following matrices:$\hat{K}$, (which captures the transition between different promoter states) whose elements are$K_{qs}=k_{q,s}$, if *q≠s* and $K_{ss}=-\sum_{q} k_{q,s}$; $\hat{R}$ is a matrix that contains the rates of initiation from different promoter states. In the case of one-step initiation it is diagonal with the diagonal elements being the rates of initiation from different promoter states i.e. $R_{sq}=k_{s,ini}\delta_{s,q}$ . In the case of two-step initiation this matrix is off-diagonal owing to the fact that the promoter state changes after initiation. For instance if the promoter switches from the *s*-th to the *q*-th state after initiation, then the off-diagonal term that exists is, $R_{qs}=k_{s,ini}$. $\hat{\Gamma}$, which captures the hopping process of RNAP molecules from one base to the next**,** is diagonal and the corresponding matrix elements are$\Gamma_{sq}=k \delta_{s,q}$. In the steady state the left hand side of equation (5) is equal to zero.

It is to be noted that for if we consider the termination rate of an RNAP molecule from the last base to be different than the hopping rate *k*. If the rate of termination is *k_term_*, then the last term of equation (5) will be different and the equation will look like the following,

$$\begin{matrix} \frac{d\vec{P}(m_{1},...,m_{L})}{dt}=(\hat{K}-\hat{R}-\hat{\Gamma}\sum_{i=1}^{L} m_{i}) \vec{P}(m_{1},.,\mathrm{m}_{i},.,m_{L})+\hat{R} \vec{P}(m_{1}-1,...,m_{L}) \\ +\sum_{i=1}^{L-1} k (m_{i}+1) \hat{\Gamma} \vec{P}(m_{1},.,m_{i}+1,m_{i+1}-1,.,m_{L}) + k_{term}(m_{L}+1) \hat{\Gamma} \vec{P}(m_{1},...,m_{L}+1). \end{matrix}$$

For the sake of simplicity we don’t incorporate the termination rate for the subsequent analysis. However, even if we explicitly use the termination rate, the analysis will remain the same.

**Mean of the nascent RNA distribution**

From equation (3), the mean number $\left\langle M\right\rangle$ of nascent RNAs along the gene is given by

$\left\langle M\right\rangle=\sum_{i=1}^{L} \left\langle m_{i}\right\rangle.$ (7)

To compute the mean of the steady-state probability distribution of nascent RNAs along the gene, we multiply both sides of equation (5) by *m_i_*, and sum over all values of *m_i_* from 0 to $\infty$. We obtain two separate equations for $\left\langle m_{1}\right\rangle$ and $\left\langle m_{i}\right\rangle$, where *i* goes from *2* to *L.*

For $m_{1}$ we find:

$\begin{matrix} \sum_{m_{1},...,m_{L}=0}^{\infty} m_{1}(\hat{K}-\hat{R}-\hat{\Gamma}\sum_{i=1}^{L} m_{i}) \vec{P}(m_{1},.,m_{i},.,m_{L})+\sum_{m_{1},...,m_{L}=0}^{\infty} m_{1}\hat{R} \vec{P}(m_{1}-1,...,m_{L}) \\ +\sum_{m_{1},...,m_{L}=0}^{\infty} \sum_{i=1}^{L} m_{1} k (m_{i}+1) \hat{\Gamma} \vec{P}(m_{1},.,m_{i}+1,m_{i+1}-1,.,m_{L})=0 . \end{matrix}$ (7)

Since none of the three matrices in equation (7) ($\hat{K}$, $\hat{R}$and $\hat{\Gamma}$) are functions of *m_1_*, they can be taken out of the sums. Simplifying the above equation further we find:

$\begin{matrix} \hat{K} \sum_{m_{1},...,m_{L}=0}^{\infty} m_{1} \vec{P}(m_{1},...,m_{L})-\hat{R} \sum_{m_{1},...,m_{L}=0}^{\infty} m_{1} \vec{P}(m_{1},...,m_{L})+\hat{R} \sum_{m_{1},...,m_{L}=0}^{\infty} m_{1} \vec{P}(m_{1}-1,...,m_{L}) \\ -\hat{\Gamma} \sum_{m_{1},...,m_{L}=0}^{\infty} m_{1}^{2} \vec{P}(m_{1},...,m_{L})-\hat{\Gamma} \sum_{i=2}^{L} \sum_{m_{1},...,m_{L}=0}^{\infty} m_{1} m_{i} \vec{P}(m_{1},.,\mathrm{m}_{i},.,m_{L}) \\ +\hat{\Gamma} \sum_{m_{1},...,m_{L}=0}^{\infty} m_{1} (m_{1}+1) \vec{P}(m_{1}+1,m_{2}-1,..,m_{L}) \\ +\hat{\Gamma} \sum_{i=2}^{L} \sum_{m_{1},...,m_{L}=0}^{\infty} m_{1} (m_{i}+1) \overrightarrow{P}(m_{1},.,m_{i}+1,m_{i+1}-1,.,m_{L}) =0 . \end{matrix}$ (8)

The equation above can be expressed in terms of the following partial moment vectors:

$\begin{matrix} \vec{m}_{1}^{(0)}=\sum_{m_{1},...,m_{L}=0}^{\infty} m_{1}^{0} \vec{P}(m_{1},...,m_{L}) , \\ \vec{m}_{1}^{(1)}=\sum_{m_{1},...,m_{L}=0}^{\infty} m_{1}^{1} \vec{P}(m_{1},...,m_{L}) . \end{matrix}$ (9)

These partial moment vectors are useful quantitates, as they are related to the moments of the probability distribution of *m_1_*. For example, the mean number of nascent RNAs at the first base is given by:

$\left\langle m_{1}\right\rangle=\sum_{s=1}^{N} \sum_{m_{1},...,m_{L}=0}^{\infty} m_{1}\text{ }p(s,m_{1},...,m_{L})=\sum_{m_{1},...,m_{L}=0}^{\infty} m_{1}\text{ }p(1,m_{1},...,m_{L})+...+\sum_{m_{1},...m_{L}=0}^{\infty} m_{1}\text{ }p(N,m_{1},...,m_{L})\text{ }.$ (10)

We simplify equation (8) and express it in terms of the partial moment vectors, defined above. In order to do that we re-arrange the third, sixth and seventh term using the fact that *m_i_*’s are dummy variables. We also use the fact that the number of nascent RNA molecules at different bases can never fall below 0 (i.e.$\vec{p} (m_{1},.,m_{i}=-1,.,m_{L})=0$, where *i* goes from 1 to *L*)$\vec{p}_{(-1)}=0)$. Using equation (8) and equation (9) we obtain equations for the two partial moment vectors:

$\begin{matrix} \hat{K} \vec{m}_{1}^{(0)}=0 , \\ (\hat{K}-\hat{\Gamma}) \vec{m}_{1}^{(1)}+\hat{R} \vec{m}_{1}^{(0)}=0 . \\ \end{matrix}$ (11) We can compute the mean of *m_1_*, by multiplying equation (11) with $\widehat{u}=(1,1,...,1)$:

$\left\langle m_{1}\right\rangle=\frac{\vec{r}\text{ }\vec{m}_{1}^{(0)}}{k} .$ (12)

Here the vector $\vec{r}$ contains the ordered list of rates of transcription initiation from each promoter state. To compute the mean of the nascent RNA number at the other bases we multiply equation (5) by *m_i_ ,* where *i* goes from *2* to *L*.

$\begin{matrix} \hat{K} \sum_{m_{1},...,m_{L}=0}^{\infty} m_{i}\vec{P}(m_{1},.,m_{i},.,m_{L})-\hat{R} \sum_{m_{1},...,m_{L}=0}^{\infty} m_{i} \vec{P}(m_{1},...,m_{L})+\hat{R} \sum_{m_{1},...,m_{L}=0}^{\infty} m_{i} \vec{P}(m_{1}-1,...,m_{L}) \\ -\hat{\Gamma} \sum_{m_{1},...,m_{L}=0}^{\infty} m_{i}^{2} \vec{P}(m_{1},.,m_{i},.,m_{L})-\hat{\Gamma} \sum_{i\neq j} \sum_{m_{1},...,m_{L}=0}^{\infty} m_{i} m_{j} \vec{P}(m_{1},.,m_{i},m_{j},.,m_{L}) \\ +\hat{\Gamma} \sum_{m_{1},...,m_{L}=0}^{\infty} m_{i} (m_{i}+1) \vec{P}(m_{1},.,m_{i}+1,m_{i+1}-1.,m_{L}) \\ +\hat{\Gamma} \sum_{i\neq j} \sum_{m_{1},...,m_{L}=0}^{\infty} m_{i} (m_{j}+1) \vec{P}(m_{1},.,m_{i},.,m_{j}+1,m_{j+1}-1,.,m_{L})=0 . \end{matrix}$ (13)

As before, we define the following partials moment vectors for convenience:

$\begin{matrix} \vec{m}_{i-1}^{(1)}=\sum_{m_{1},...,m_{L}=0}^{\infty} m_{i-1}^{1} \vec{P}(m_{1},.,\mathrm{m}_{i-1},.,m_{L}) , \\ \vec{m}_{i}^{(1)}=\sum_{m_{1},...,m_{L}=0}^{\infty} m_{i}^{1} \vec{P}(m_{1},.,\mathrm{m}_{i},.,m_{L}) . \end{matrix}$ (14)

Following the same procedure as *m_1_*, we can find the mean of *m_i_* from equation (13), in terms of the partial moments (equation (14)) of *m_i_* and *m_i-1_*:

$(\hat{K}-\hat{\Gamma}) \vec{m}_{i}^{(1)}+\hat{\Gamma} \vec{m}_{i-1}^{(1)}=0 .$ (15)

We compute the mean of *m_i_*, by multiplying equation (15) with $\widehat{u}=(1,1,...,1)$. The transition matrix $\hat{K}$has the property that the sum of the elements of any one of its columns is always 0. Using this property, we find that, $\vec{u}\text{ }\hat{K}=0$. Therefore, we find that the mean nascent RNA number at each base is the same as the mean of the previous one, i.e.,

$\left\langle m_{i}\right\rangle=\left\langle m_{i-1}\right\rangle.$ (16)

Using equation (14) and equation (15) we get:

$\left\langle m_{L}\right\rangle=\left\langle m_{L-1}\right\rangle=...=\left\langle m_{1}\right\rangle.$ (17)

In other words in the steady state, the mean number of nascent RNAs at every base along the gene is the same. Hence the mean nascent RNA number$\left\langle M\right\rangle$ along the gene is the sum of the mean of nascent RNA numbers at each base. Therefore from equation (3), equation (11) and equation (16), we find that the mean is proportional to the length of the gene:

$\left\langle M\right\rangle=L\frac{\vec{r} \vec{m}_{1}^{(0)}}{k} .$ (18)

For instance, using this formula above one can find the mean number of nascent RNAs along a gene which initiates transcription in uncorrelated events at a constant rate *r*. For such a gene the mean number of nascent RNAs is given $\left\langle M\right\rangle=L\frac{r }{k} .$ This result can be understood from a simple consideration. Namely, *L/k* is the mean time that an RNAP spends on the gene before completing transcription. Since *r* is the rate at which RNAPs enter the elongation state, the mean number of RNAPs on the gene at any time is the product of the two.

**Variance of the nascent RNA distribution**

In order to compute the variance, we need to find all the elements of the covariance matrix of the dependent random variables, (*m_1,…_m_L_*). Using equation (3), we find that the second moment is:

$$\left\langle M^{2}\right\rangle=\sum_{i=1}^{L} \left\langle m_{i}^{2}\right\rangle+2 \sum_{j\neq i} \left\langle m_{i} m_{j}\right\rangle.$$

We expand it for convenience:

$\left\langle M^{2}\right\rangle=\left\langle m_{1}^{2}\right\rangle+2 \left\langle m_{1} m_{2}\right\rangle+2 \sum_{i=3}^{L} \left\langle m_{1} m_{i}\right\rangle+\sum_{i=2}^{L} \left\langle m_{i}^{2}\right\rangle+2 \sum_{i=2}^{L-1} \left\langle m_{i} m_{i+1}\right\rangle+2 \sum_{j=i+2}^{L} \sum_{i=2}^{L-2} \left\langle m_{i} m_{j}\right\rangle.$ (19) To find $\left\langle M^{2}\right\rangle$, we need to compute each term on the right hand side using equation (5). As before we define the following partial moments:

$\begin{matrix} \vec{m}_{i}^{(2)}=\sum_{m_{1},...,m_{L}=0}^{\infty} m_{i}^{2} \vec{P}(m_{1},...,m_{L}) , \\ \vec{m}_{i,j}^{(2)}=\sum_{\begin{matrix} m_{1},...,m_{L}=0 \\ \end{matrix}}^{\infty} m_{i}^{1} m_{j}^{1} \vec{P}(m_{1},.,m_{i},.,m_{j},.,m_{L}) . \end{matrix}$ (20)

Here *i* and *j* both range from 1 to *L.* We get a set of six equations for the partial moments defined above by multiplying equation (5) with *m_1_^2^*, *m_1_m_2_* , *m_1_m_i_* (where *i* goes from 3 to *L*)*, m_i_^2^*

(where *i* goes from 2 to *L*), *m_i_m_i+1_* (where *i* goes from 2 to *L-1*) and *m_i_m_j_* (where *i* goes from *2* to *L-2* and *j* runs from *i+2* to *L)*

$(\hat{K}-2\hat{\Gamma}) \vec{m}_{1}^{(2)}+2\hat{R} \vec{m}_{1}^{(1)}+\hat{\Gamma} \vec{m}_{1}^{(1)}+\hat{R} \vec{m}_{1}^{(0)}=0 .$ (21)

(22)

$(\hat{K}-2\hat{\Gamma}) \vec{m}_{1,i}^{(2)}+\hat{\Gamma} \vec{m}_{1,i-1}^{(2)}+\hat{R} \vec{m}_{3}^{(1)}=0 .$ (23)

$(\hat{K}-2\hat{\Gamma}) \vec{m}_{i}^{(2)}+2\hat{\Gamma} \vec{m}_{i,i-1}^{(2)}+\hat{\Gamma} (\vec{m}_{i}^{(1)}+\vec{m}_{i-1}^{(1)})=0 .$ (24)

$\begin{matrix} (\hat{K}-2\hat{\Gamma}) m_{i,i+1}^{(2)}+\hat{\Gamma} \vec{m}_{i-1,i+1}^{(2)}+\hat{\Gamma} (\vec{m}_{i}^{(2)}-\vec{m}_{i}^{(1)})=0 . \\ \end{matrix}$ (25) $(\hat{K}-2\hat{\Gamma}) \vec{m}_{i,j}^{(2)}+\hat{\Gamma} \vec{m}_{i,j-1}^{(2)}+\hat{\Gamma} m_{i-1,j}^{(2)}=0 .$ (26)

From equation (21-26), we can compute the second order moments for the nascent RNA numbers at every base along the gene by multiplying the partial moment vectors with $\widehat{u}=(1,1,...,1)$. In order to obtain the variance, we construct the covariance matrix, whose element in the *i*, *j* position is the covariance between the random variables characterizing the number of nascent RNAs at the  *i*-th and *j*-th bases along the gene, defined as

$\text{Covariance} (m_{i}m_{j})=\left\langle m_{i}m_{j}\right\rangle-\left\langle m_{i}\right\rangle\left\langle m_{j}\right\rangle=\left\langle m_{i}m_{j}\right\rangle-\left\langle m_{1}\right\rangle^{2} .$ (27)

Here we use the fact that $\left\langle m_{i}\right\rangle=\left\langle m_{j}\right\rangle=\left\langle m_{1}\right\rangle$. The dimension of the covariance matrix is $L\times L$ and since the number of bases *L* along a gene is typically of the order of few thousands, the number of second order moments to be evaluated to construct the covariance matrix is very high. However, it is readily evaluated with Mathematica using equation (21-26) and equation (27). Therefore the variance of the number of nascent RNA molecules along a gene is given by

$\text{Variance}=\left\langle M^{2}\right\rangle-N^{2}\left\langle m_{1}\right\rangle^{2},$ (28)

which is just the sum of all the elements of the covariance matrix.

**Supplementary Figures**


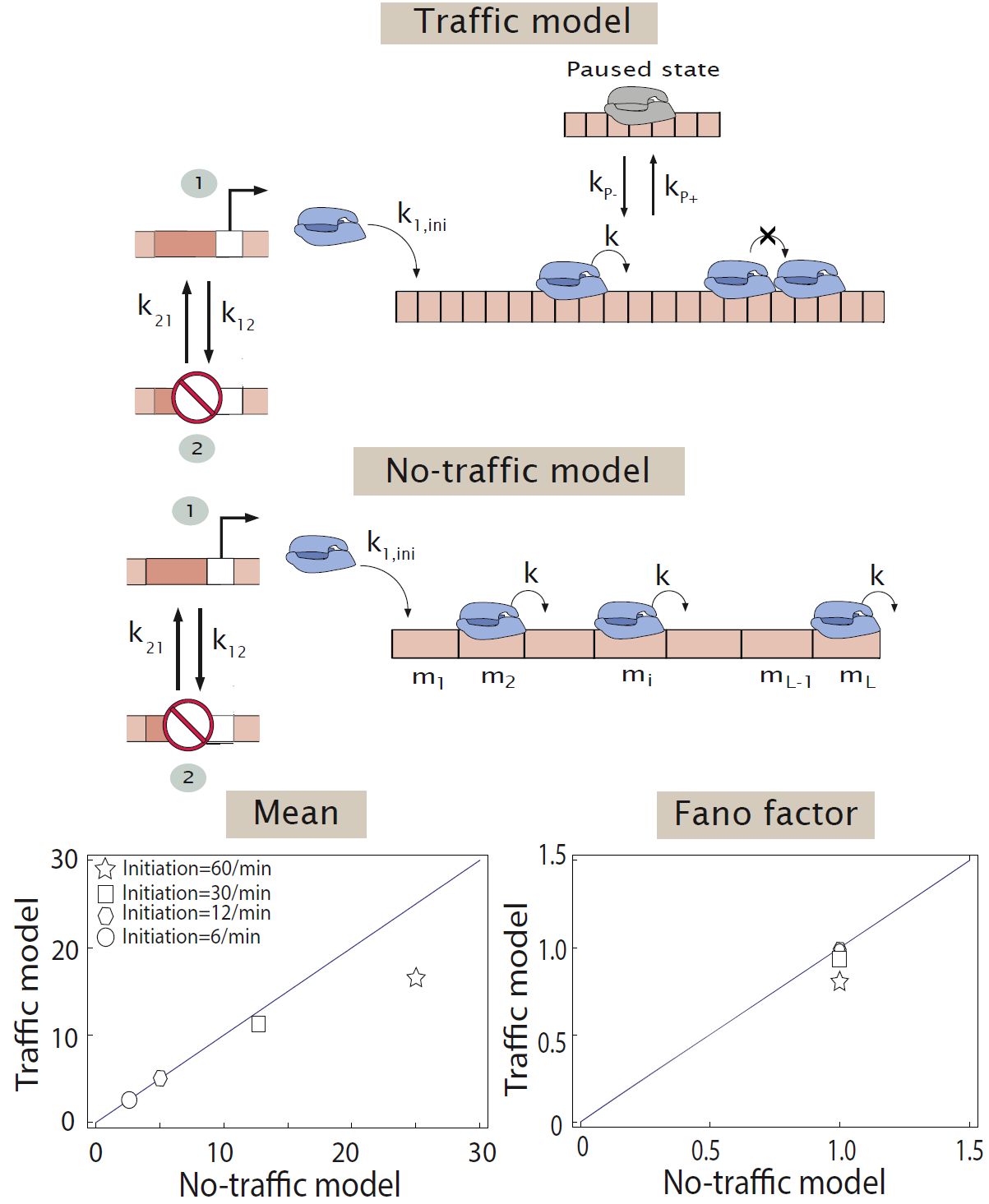


Figure 1: (A) **Two state promoter with traffic**. The promoter switches between two states: state *1*, from which transcription initiation occurs with a constant probability per unit time *k_1,ini_*, and state 2, from which transcription does not initiate. The promoter switches from state 1 to state 2 with probability per unit time *k_12_*, and from 2 to 1 with probability per unit time *k_21_*. After initiation each RNAP molecule hops from one base pair to the next along the gene at a rate *k* per unit time. Each RNAP molecule has a finite DNA footprint of *30 bp* and it can pause at any site with a rate *k_P+_* and come out of the pause with a rate *k_P-_*. An RNAP molecule cannot move forward if another one occupies the bases in front of it. The length of the gene is *L.* (B) **No-traffic model.** The elongation process is uniform with each RNAP molecule occupying one base pair. (C) Using the Gillespie algorithm [5,6], we simulate the traffic model where the promoter initiates at a constant rate, where we take four different values of initiation. Predictions of the no-traffic model for the mean and Fano factor agree well with the simulation results from the traffic model up to an initiation rate of 30 initiations/min. The elongation parameters used, are *k_P-_=* 4/sec, *k_P+_=*0.01/sec, *k=*80 bp/sec [7] as reported for ribosomal promoters in *E.Coli*. The length of the gene is *L*= 2000 bps and the footprint of one RNAP molecule is 30 bps.


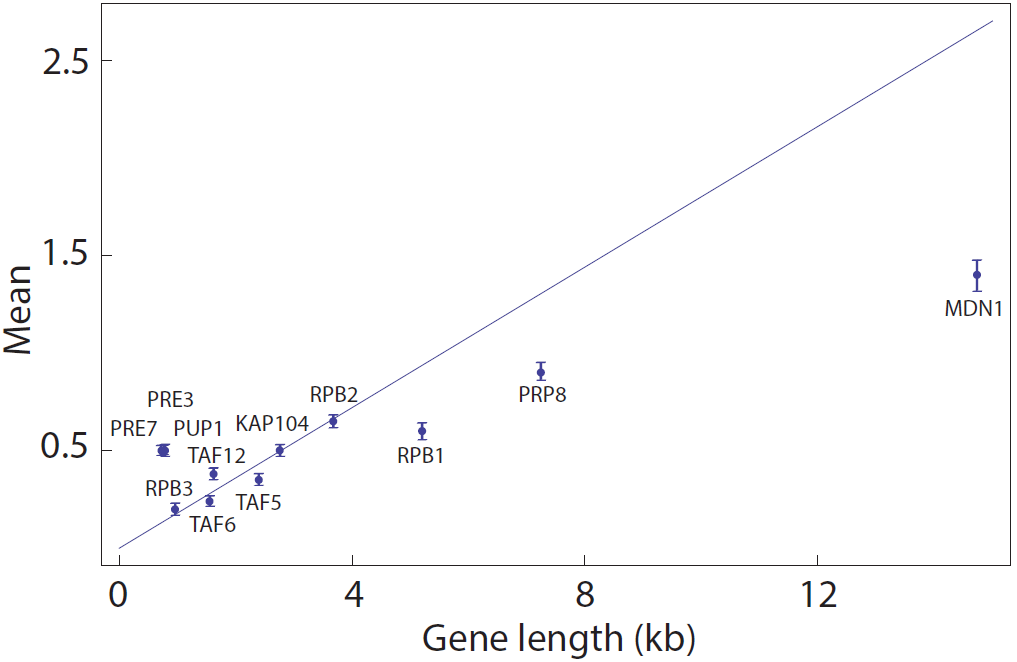


Figure 2: **Mean number of nascent RNAs in yeast.** Here we plot the mean of the nascent RNA distribution for all twelve genes: MDN1, PRP8, RPB1, PUP1, PRE3, PRE7, KAP104, TAF5, TAF6, TAF12, RPB2, RPB3, reported by Gandhi et al. [8]. For six of these twelve genes, KAP104, TAF5, TAF6, TAF12, RPB2, RPB3, the mean increases linearly with the gene length, as shown in the main text in Fig. 2A. The other six genes do not follow the same trend line indicating that they have different initiation rates; PUP1, PRE3, PRE7, have similar initiation rates but different from the six genes analyzed in the main text.


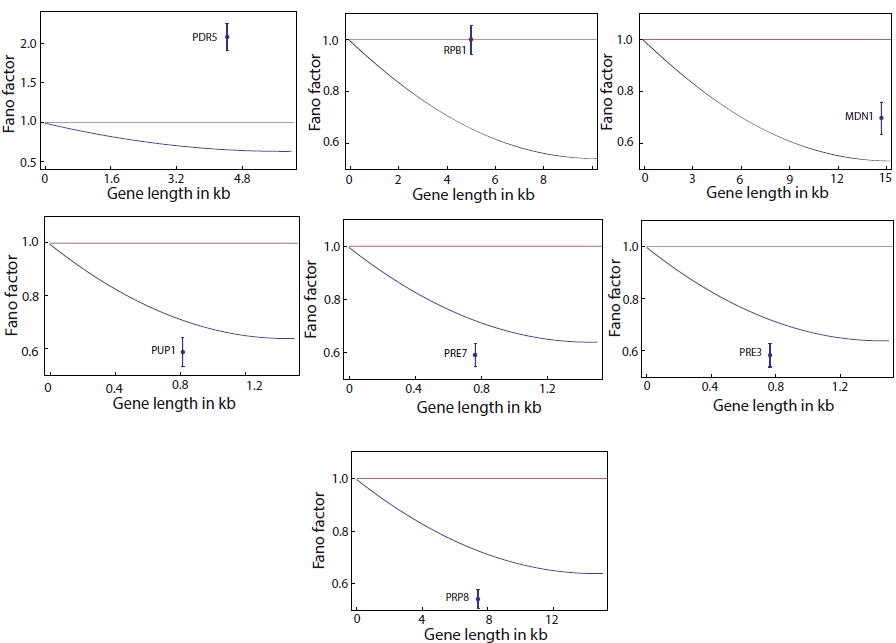


Figure 3**: Fano factor for nascent RNA distributions in yeast.** Here we show 6 different constitutively expressed genes in yeast [8]: RPB1, MDN1, PUP1, PRE7, PRE3, PRP8 which have different initiation rates than the six genes shown in Figure 2, and another gene PDR5, known to be regulated [9]. The data for Fano factors measured in experiments are shown in comparison with the predictions for the one-step (red line) and two-step (area between red line and blue line) initiation models. The Fano factor for the PDR5 gene is greater than one, which is consistent with an ON-OFF model of transcription initiation. RPB1 has a Fano factor equal to 1, suggesting one-step initiation. All the other genes have Fano factors consistent with more than one rate-limiting step leading up to initiation. The blue line indicates the minimum possible Fano factor for the specific gene assuming two-step initiation and the measured average initiation rate, which we compute from the mean number of nascent RNAs. For the genes whose Fano factor is below this line (PUP1, PRE7, PRE3, PRP8) the data suggest that their transcription is initiated via three or more (similar in duration) steps.

**References**

1. Sanchez A, Choubey S, Kondev J (2013) Regulation of Noise in Gene Expression. Annu Rev Biophys 42: 469–491. doi:10.1146/annurev-biophys-083012-130401.

2. Sanchez A, Choubey S, Kondev J (2013) Stochastic models of transcription: From single molecules to single cells. Methods San Diego Calif 62: 13–25. doi:10.1016/j.ymeth.2013.03.026.

3. Sanchez A, Garcia HG, Jones D, Phillips R, Kondev J (2011) Effect of promoter architecture on the cell-to-cell variability in gene expression. PLoS Comput Biol 7: e1001100. doi:10.1371/journal.pcbi.1001100.

4. Kepler TB, Elston TC (2001) Stochasticity in transcriptional regulation: origins, consequences, and mathematical representations. Biophys J 81: 3116–3136.

5. Gillespie DT (1976) A general method for numerically simulating the stochastic time evolution of coupled chemical reactions. J Comput Phys 22: 403–434. doi:10.1016/0021-9991(76)90041-3.

6. Gillespie DT (1977) Exact stochastic simulation of coupled chemical reactions. J Phys Chem 81: 2340–2361. doi:10.1021/j100540a008.

7. Klumpp S, Hwa T (2008) Stochasticity and traffic jams in the transcription of ribosomal RNA: Intriguing role of termination and antitermination. Proc Natl Acad Sci 105: 18159–18164. doi:10.1073/pnas.0806084105.

8. Gandhi SJ, Zenklusen D, Lionnet T, Singer RH (2011) Transcription of functionally related constitutive genes is not coordinated. Nat Struct Mol Biol 18: 27–34. doi:10.1038/nsmb.1934.

9. Zenklusen D, Larson DR, Singer RH (2008) Single-RNA counting reveals alternative modes of gene expression in yeast. Nat Struct Mol Biol 15: 1263–1271. doi:10.1038/nsmb.1514.
